# Phosphorylation of RpoD upregulates suilysin and enhances *Streptococcus suis* virulence

**DOI:** 10.1186/s13567-026-01864-1

**Published:** 2026-10-30

**Authors:** Yutong Tian, Zixuan Zhao, Hang Yin, Xinghong Liu, Jiaxiang Fu, Letao Gao, Yuqing Li, Rendong Fang, Lianci Peng

**Affiliations:** Joint International Research Laboratory of Animal Health and Animal Food Safety, College of Veterinary Medicine, Southwest University, No.2 Tiansheng Road, Beibei District, Chongqing, 400715 China

**Keywords:** *Streptococcus suis*, STK, SLY, transcription factor, virulence

## Abstract

*Streptococcus suis* (*S. suis*) is a prevalent zoonotic pathogen that severely threatens the swine industry and human public health. Suilysin (SLY), a major pore-forming toxin of *S. suis*, is indispensable for bacterial hemolytic activity and systemic pathogenicity, yet the upstream regulatory mechanism controlling SLY production remains unclear. Serine/threonine kinase (STK) has been characterized to modulate bacterial physiology and virulence by phosphorylating multiple substrate proteins. Our previous phosphoproteomics analysis indicated that the housekeeping sigma factor RpoD was a potential STK substrate. In vitro phosphorylation assays combined with mass spectrometry, co-immunoprecipitation and site-directed mutagenesis confirmed that STK directly phosphorylated RpoD at Thr-208 residue. Phenotypic tests revealed that RpoD phosphorylation had no obvious impact on bacterial growth and biofilm formation but specifically promoted the thermotolerance and hemolytic activity of *S. suis*. Notably, the *rpoD* overexpression mutant strain SC19-rpoD significantly upregulated the transcription and protein production of SLY, whereas the phosphorylation-deficient mutant SC19-rpoD T208A abolished this promotion effect, indicating that Thr-208 phosphorylation is required for *sly* upregulation, although the underlying molecular mechanism remains to be fully defined. Furthermore, mouse infection models demonstrated that the SC19-rpoD T208A mutant exhibited alleviated splenic pathological injury, reduced bacterial loads in multiple organs, and significantly improved host survival, indicating that the loss of RpoD phosphorylation impairs *S. suis* virulence. Collectively, these findings demonstrate that STK-mediated phosphorylation of RpoD at Thr-208 is required for *sly* upregulation and contributes to the *S. suis* pathogenesis.

**Supplementary Information:**

The online version contains supplementary material available at https://doi.org/10.1186/s13567-026-01864-1.

## Introduction

*Streptococcus suis* (*S. suis*) is a major zoonotic pathogen that causes meningitis, septicemia, and arthritis in pigs and humans, posing a significant threat to global public health [1–3]. The pathogenicity of *S. suis* is governed by a diversity of virulence factors that collectively mediate bacterial colonization, immune evasion, and host tissue damage, leading to its complex pathogenic mechanisms [4–6]. Streptococcal toxic shock syndrome (STSS) is one of the most classic syndromes [7], which is triggered by inflammatory storms [8, 9]. Suilysin (SLY), one of the most abundant pore-forming toxins of *S. suis*, plays a critical role in bacterial infection and the inflammatory response [10]. Therefore, SLY has emerged as a promising therapeutic target, and the inhibition of SLY activity has become an effective anti-*S. suis* strategy [11]. Several natural products, such as acacetin [6], quercetin [12], silibinin [13], and myricetin [14] have been reported to alleviate *S. suis* infection by inhibiting SLY activity. The pathogenic role of SLY in the host has been well characterized, while the regulatory mechanism that modulates SLY remains poorly understood.

Serine/threonine kinase (STK)-mediated phosphorylation regulates bacterial gene transcriptional profiles, adaptation to the host immune stress, virulence, and antibiotic tolerance [15]. In *S. suis*, STK has been verified to phosphorylate different substrates, such as cell division initiation protein (DivIVA) [16], capsule control protein S (CcpS) [17], fatty acid degradation regulator (FadR) [18], phosphoglucosamine mutase (GlmM) [19], translation elongation factor P (EF-P) [20], and *Streptococcus suis* Ser/Thr kinase phosphorylation substrate 1 (SstP1) [1], thereby regulating bacterial growth, capsular polysaccharide synthesis, biofilm formation, stress resistance, and pathogenicity. RpoD, the primary sigma factor, plays a critical role in the transcription and expression of majority of bacterial genes [21–23]. Our previous phosphoproteomic analysis revealed that RpoD is a potential substrate of STK. Nevertheless, it remains unclear whether this phosphorylation event alters biological functions of RpoD, thereby modulating bacterial virulence.

In the present study, we identified RpoD as a novel substrate of STK in *S. suis* and confirmed its phosphorylated site at Thr208. This modification upregulates *sly* transcription. In addition, we further explored the effects of this modification on bacterial virulence both in vitro and in vivo. Our findings reveal a novel STK–RpoD–SLY regulatory axis, which provides new insights into the pathogenic mechanism of *S. suis* and potential targets for the development of anti-*S. suis* strategies.

## Materials and methods

### Ethics statements

Female wild-type (WT) C57BL/6 mice were purchased from Chongqing Enswell Biotechnology Co., Ltd. All mice were maintained under specific pathogen-free (SPF) conditions before being used at 8–10 weeks of age. All the animal experiments were approved by the Southwest University Ethics Committee, Chongqing, China (IACUC-20231215–02).

### Plasmid construction

The plasmids pSET2-rpoD and pSET2-rpoD-T208A were used for *S. suis* SC19 mutant strain construction, resulting in SC19-rpoD and SC19-rpoD T208A mutants, respectively. The coding sequences of *rpoD* and *stk* were polymerase chain reaction (PCR)-amplified with specific primers and ligated into the pET28a and pGEX vectors, respectively, to generate polyhistidine (His)- and glutathione S-transferase (GST)-tagged fusion constructs (pET28a-rpoD and pGEX-stk) for protein expression. The mutant plasmids, including RpoD-S93A, RpoD-T94A, and RpoD-T208A, were obtained from BGI (Beijing, China). These mutant plasmids were inserted into pET28a vector for subsequent mutant protein expression.

### Bacterial strains

The *S. suis* epidemic strain SC19 (serotype 2) was used in the present study. SC19 and the mutants were cultured in Todd–Hewitt broth (THB) or agar (THA) supplemented with 10% fetal bovine serum (FBS) at 37 °C. *E. coli* was cultured in lysogeny broth (LB) or LB supplemented with 1.5% agar. For plasmid maintenance, kanamycin (50 μg/mL) was added to the media of *E. coli* harboring pET28a, and spectinomycin (100 μg/mL) was added to *S. suis* harboring pSET2.

### * Streptococcus suis* mutant strain construction

The constructed plasmids pSET2-rpoD and pSET2-rpoD T208A were electroporated into SC19 competent cells and cultured at 37 °C for 12 h to generate SC19-rpoD and SC19-rpoD T208A mutants. Electroporation was conducted in the THB culture medium by a Bio-Rad Gene Pulser Xcell system with 0.1 cm cuvettes (2 kV, 1000 Ω, and 25 μF). These constructed mutant strains were stored in our laboratory.

### Cell culture

The human cerebral microvascular endothelial cell line (hCMEC/D3, JNO-H0520, Jennio Biotech) cells were maintained in Dulbecco’s modified eagle medium (DMEM; Gibco, C11995500BT) supplemented with 10% FBS (ExCell Bio, FSP500) at 37 °C in a humidified incubator with 5% CO_2_.

### Bacterial growth curve

Bacterial suspensions of WT SC19, SC19-rpoD, and SC19-rpoD T208A were incubated in THB supplemented with 10% FBS at 37 °C with continuous shaking for 0, 1, 2, 3, 4, 6, and 8 h. After incubation, the absorbance of the bacterial suspension at 600 nm was measured using a spectrophotometer.

### Protein expression and purification

The primers used in the amplification of target DNA fragments in the present study are listed in Additional file 1. The coding sequences of *stk* and *rpoD* were amplified from the SC19 genome via the primers nSTK-F/nSTK-R and rpoD-F/rpoD-R (including rpoD mutants), respectively. The PCR products of nSTK and rpoD were then each inserted into the digested pGEX-4 T and pET28a vector, respectively. Subsequently, vectors were electroporated into BL21 competent cells. To induce protein expression, BL21 harboring pGEX-nSTK and pET28a-rpoD vectors with GST and His-tagged constructs, respectively, were grown to an optical density (OD)600 of 0.8 in LB at 37 °C with shaking at 180 rpm and then induced with isopropyl β-D-1-thiogalactopyranoside (IPTG) at a final concentration of 1 mM at 37 °C for 4 h with shaking at 180 rpm. Protein purification was performed using Ni Elite Beads (Vazyme, Nanjing, China) and BeyoGold™ GST-tag purification resin (Beyotime, China) for His-tagged and GST-tagged proteins, respectively. Finally, His-rpoD and GST-STK proteins were obtained.

### Polyclonal antibody preparation

C57BL/6 mice were subcutaneously immunized with 100 μg of purified His-RpoD emulsified in adjuvant three times at 0, 14, and 28 days. The serum was collected 36 days after immunization.

### In vitro phosphorylation assay

To detect the phosphorylation of STK and RpoD, 50 μg of purified recombinant RpoD protein or 150 μg of purified STK protein were incubated in 50 μL of phosphorylation buffer (100 mM Tris–HCl pH 8.0, 10 mM MgCl_2_, 25 mM NaCl, 10 mM ATP, and 1 mM DTT) or in nonphosphorylation buffer for 2 h at 37 °C. Phosphorylation buffer was procured on ice. Then, sodium dodecyl sulfate (SDS) loading buffer (5×) was added, and the samples were boiled at 95 °C for 5 min. To exclude the effects of autophosphorylation of STK and RpoD, STK or RpoD alone was added as control. Next, samples were separated on 8% Tris–glycine sodium dodecyl sulfate–polyacrylamide gel electrophoresis (SDSPAGE) gels and 8% Phos-tag™ gels containing 100 μM Phos-tag™ acrylamide and 200 μM MnCl_2_ (Vazyme, Nanjing, China), which specifically retards the migration of phosphorylated proteins. Finally, the proteins were visualized by coomassie brilliant blue R-250 staining for 1 h, followed by destaining (40% methanol, 10% acetic acid) for 2‒4 h.

### Immunoblot analysis of RpoD phosphorylation

SC19, *Δstk*, and C*Δstk* were grown to the mid-logarithmic phase with an OD of 0.6, and bacteria were harvested by centrifugation at 4000 × *g* for 10 min at 4 °C. The bacteria were subsequently washed twice with ice-cold phosphate-buffered saline (PBS) and resuspended in 1 mL of a PBS containing lysozyme (20 mg/mL) and protease inhibitor cocktail (Beyotime, China). Next, the samples were sonicated on ice using a 3-mm probe at 30% amplitude for ten cycles (10 s pulse, 10 s rest). Lysates were centrifugated at 12000 × *g* for 15 min at 4 °C and supernatants were collected. The protein concentration was determined with a bicinchoninic acid (BCA) protein assay kit. The total protein (1 mg) and anti-RpoD polyclonal antibodies were incubated with Protein A + G agarose (Beyotime, China) overnight at 4 °C. The beads were washed three times with 1 mL of ice-cold radio-immunoprecipitation assay (RIPA) buffer (50 mM Tris–HCl pH 8.0, 150 mM NaCl, 1% NP-40, and 0.5% sodium deoxycholate). The bound proteins were eluted by boiling in 2 × Laemmli buffer (65 mM Tris–HCl pH 6.8, 2% SDS, 10% glycerol, and 5% β-mercaptoethanol) at 95 °C for 10 min.

For detecting STK-induced phosphorylation of RpoD and its mutants, purified His-RpoD, His-RpoD-S93A, His-RpoD-T94A, or His-RpoD-T208A (50 μg) was incubated with or without purified GST-STK (150 μg) in 50 μL phosphorylation buffer (100 mM Tris–HCl pH 8.0, 10 mM MgCl_2_, 25 mM NaCl, 10 mM ATP, and 1 mM DTT) at 37 °C for 2 h. The reaction was terminated by adding 5 × SDS loading buffer and boiling at 95 °C for 5 min.

Samples and markers (10-250 kDa, Vazyme, China) were subjected to sodium dodecyl sulfate polyacrylamide gel electrophoresis (SDS-PAGE) and ran at 80 V for 30 min, 120 V for 70 min, and subsequently transferred onto a polyvinylidene difluoride (PVDF) membrane by electroblotting at 250 mA for 90 min. Next, the membranes were blocked with 5% nonfat dry milk and then immunoblotted with the primary antibodies including anti-RpoD (1:1000), anti-His (Protech 66,005–1-Ig, 1:5000), and anti-phospho-(Ser/Thr) (Abcam ab17464, 1:2000), overnight at 4 °C. The next day, the blots were incubated with horseradish peroxidase-conjugated goat anti-mouse/rabbit immunoglobulin G (IgG) (1:2000 Beyotime, China). Finally, the distinct protein bands were detected with SuperKine™ West Femto maximum sensitivity substrate (Abbkine, China) and visualized in a ChemiDoc Imaging System (Bio-Rad).

### Multiple sequence alignment

RpoD sequences of different bacteria were downloaded from National Center for Biotechnology Information (NCBI). Multiple sequence alignments of RpoD orthologs were performed using Clustal Omega, and the conserved Thr-208 containing region was visualized.

### RNA extraction, reverse transcription polymerase chain reaction (RT-PCR), and quantitative PCR (qPCR)

Total RNA was isolated using the bacterial RNA isolation kit (Vazyme, China) and reverse transcribed into complementary DNA (cDNA) using HiScript Q Select RT SuperMix (Vazyme, China) according to the manufacturer’s instructions. Then, qPCR was performed to verify the successful gene deletion, or site-directed mutagenesis, and *sly* gene expression (qPCR primers: sly-F: ACCTGCATCCCCACCAAAAA, sly-R: TACCCAAGTTCAAGCCGCAT, gapdh-F: GCAATATCAGCCTTGCCATCGT, and gapdh-R: GCGACTGAACTTGGTGCAAAAG). The relative expression levels were calculated using the 2^(−ΔΔCT)^ method.

### Detection of biofilm formation

WT SC19, SC19-rpoD, and SC19-rpoD T208A strains were grown to OD_600_ of 0.6–0.8 in THB at 37 ℃ and diluted to a OD_600_ of 0.1 bacterial suspensions (1:100 dilution in fresh THB, 200 μL) at a concentration of 10^6^ colony-forming units (CFU)/mL, and were incubated in 96-well plates at 37 °C for 24 h. Each well was gently washed with PBS three times by pipetting. The remaining biofilm was fixed by methanol (200 μL) for 15 min and then stained with 0.1% crystal violet stain for 15 min. After adding 200 μL of ethanol, the absorbance was measured at 570 nm with a Tecan GENios Plus microplate reader.

### Environmental stress survival assays

Environmental stress survival assays were performed under acidic conditions of pH 5.0 for 1 h and heat stress at 42 °C for 1 h in WT SC19, SC19-rpoD, and SC19-rpoD T208A strains. After exposure, bacterial suspensions were serially diluted and plated on THB agar to incubate in a 5% CO*2* aerobic atmosphere at 37 °C for 12 h. Survival rates were counted to assess their survival.

### Hemolytic activity detection

Hemolytic activity (HA) was detected according to previously reported methods [24]. Briefly, the supernatant of bacterial suspensions were collected from the mid-exponential phase (OD_600_ = 0.6) by centrifugation at 12000 × *g* for 5 min. Each 50 μL of the supernatant was incubated with 150 μL 2% swine red blood cells for 2 h at 37 °C in a 96-well plate. Triton X-100 (0.1%) in PBS and sterile PBS were used as positive and negative controls, respectively. Then, the plate was centrifuged at 1000 × *g* for 10 min and supernatants (150 μL) were transferred to a new 96-well microplate to measure the absorbance at 550 nm. Hemolysis rate(%) = (A_sample_–A_negative_)/(A_positive_–A_negative_) × 100%.

### Survival in swine whole blood

SC19, SC19-rpoD, and SC19-rpoD T208A were cultured in THB with 10% FBS to the mid-exponential phase (OD_600_ = 0.6). Bacteria were collected and adjusted to 0.01 at OD_600_ with PBS. Subsequently, 900 μL of fresh blood was mixed with a 100 μL bacterial suspension (2.5 × 10^5^ CFUs) and incubated for 3 h at 37 °C with gentle shaking. At 3 h incubation, the mixtures were serially diluted, and plated onto THB with 10% FBS plates to determine the survival bacteria. Survival rates were calculated as follows: (recovered CFU at 3 h)/(CFU before blood killing) × 100%.

### Detection of lactate dehydrogenase (LDH)

HCMEC/D3 cells were seeded in 96-well plates and infected with SC19, SC19-rpoD, or SC19-rpoD T208A at a multiplicity of infection (MOI) of 10 for 4 h. The culture supernatants were collected for lactate dehydrogenase (LDH) release measurement using a LDH cytotoxicity assay kit (Beyotime, China) according to the manufacturer’s instructions. Briefly, 50 μL of supernatant was mixed with 50 μL of the reaction reagent and then incubated at room temperature for 30 min in the dark, followed by the addition of 50 μL stop solution. The absorbance was measured at 490 nm using a microplate reader (Bio-Rad, Japan). Cell culture medium alone was used as the background control. The level of released LDH was calculated using the standard curve. Relative LDH release = A _Sample_/A _background control_.

### Mice infection

Mice at the age of 8–10 weeks and the weight of 18–20 g were randomly divided into four groups, including PBS, SC19, SC19-rpoD, and SC19-rpoD T208A.

We used female C57BL/6 mice (Cavens Laboratory Animal Co., Ltd., Changzhou, China) as animal infection models. To minimize external interference, mice were kept in noise-control conditions, and one third of the soiled bedding was retained when changing cages. Mice at the age of 7–8 weeks and with a weight of 18–20 g were selected and randomly assigned to each experiment (a total of 101 mice were included, 10 or 3 per group). Only the personnel responsible for data analysis were aware of the group allocations. Survival assay was conducted by intraperitoneal injection with bacteria at a 2.5 × 10^8^ and 5 × 10^7^ colony-forming units (CFUs) dose. Clinical manifestations were recorded every 8 h until the completion of the infection experiments or upon the death of the individual mouse. Bacterial burden assay was performed by intraperitoneal injection with 5 × 10^7^ CFUs of bacteria. Mice were sacrificed with isoflurane at 24 h postinfection (hpi) for tissue collections. To quantify the bacterial burden in each organ, tissues were homogenized with sterile PBS and serially diluted to plate onto THB agar for CFUs counting. Pathological analysis was performed in the liver, lung, brain, and spleen by fixing in 4% paraformaldehyde (Beyotime, CN, P0099) for sectioning and hematoxylin–eosin (HE) staining. Microscopic lesion assessments were performed by evaluating five fields from each tissue section randomly and recorded as 0 (no lesions), 1 (minimal), 2 (mild), 3 (moderate), or 4 (severe) depending on the pathological conditions. The experimental animal protocol was reviewed and approved by the Southwest University Animal Ethics Committee (approval no.: IACUC-20231215-02).

### Statistical analysis

**Figure 1:**
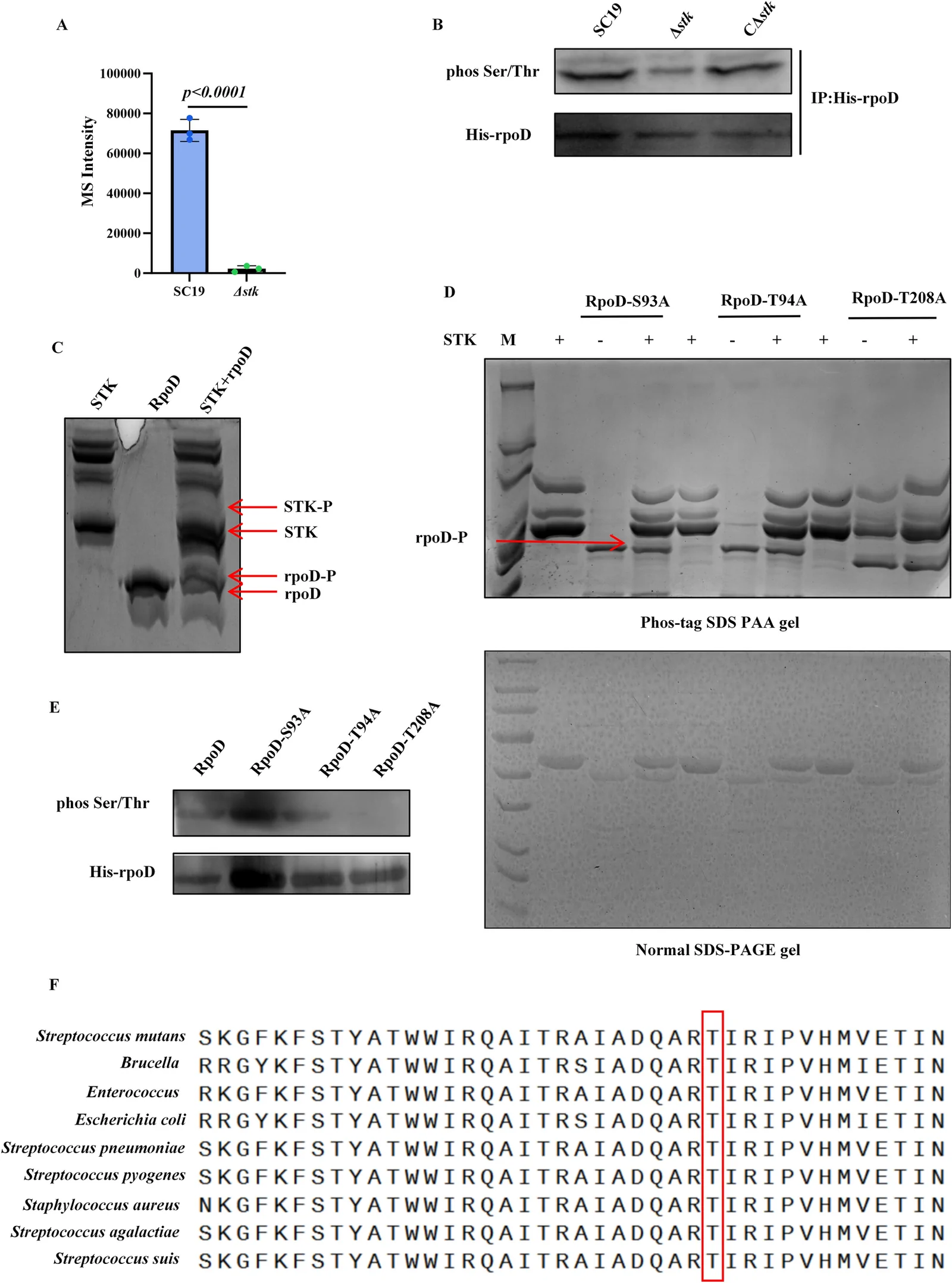
**STK directly phosphorylates rpoD at Thr-208 in** ***S. suis.*** **A** Phosphoproteomics analysis showing reduced phosphopeptide signals of rpoD in the Δ*stk* mutant compared with wild-type (WT) SC19. **B** Immunoblot analysis of rpoD phosphorylation in the lysates of WT SC19, Δ*stk,* and CΔ*stk*. **C** Phos‑tag SDS-PAGE analysis showing rpoD phosphprylation by STK using purified GST‑STK and His‑rpoD protein. **D** Phos‑tag SDS-PAGE analysis showing rpoD phosphorylation by co-incubation of purified rpoD, rpoD-S93A, rpoD-T94A, and rpoD-T208A protein with STK. **E** Immunoblot analysis of rpoD phosphorylation at T208 induced by STK in vitro by co-incubation of purified rpoD, rpoD-S93A, rpoD-T94A, and rpoD-T208A protein with STK. **F** Sequence alignment of the conserved T208 containing region in rpoD orthologs of different species. Data are representative of three independent experiments and the exact *p* values are shown in the figure.

The data are presented as the mean ± SD of at least three independent biological experiments for each group (*n* = 3, 5, or 10). One-way analysis of variance (ANOVA) was used to analyze the significant differences for comparisons among different groups. The exact *p* values are shown in Figure 1 and a value of *p* < 0.05 was considered statistically significant. Survival-curves were compared using the log-rank (Mantel–Cox) test to determine significance between the SC19-rpoD and SC19-rpoD T208A groups.

## Results

### STK phosphorylated RpoD at Thr-208 in *S. suis*

Our previous phosphoproteomics analysis [20] revealed a reduced phosphopeptide signal of RpoD in the Δ*stk* mutant compared with WT *S. suis* (Figure 1A), indicating that RpoD is a potential substrate of STK. Therefore, RpoD phosphorylation was detected in SC19, Δ*stk,* and complemented Δ*stk* (CΔ*stk*) using immunoprecipitation (IP) assay. The results showed that *stk* knockdown significantly reduced RpoD phosphorylation level, but complementary *stk* recovered its phosphorylation (*p* < 0.0001, Figure 1B). To further confirm that STK phosphorylates RpoD, in vitro phosphorylation assay in Phos-tag™ gels was performed by the co-incubation of STK and RpoD proteins. Compared with RpoD alone, RpoD with STK exhibited a migratory band (Figure 1C). Next, RpoD phosphorylation sites were identified by mass spectrometry analysis. The results showed that RpoD phosphorylation occurred on three threonine residues at Thr-93, Ser-94, and Thr-208. Therefore, we expressed the RpoD-S93A, RpoD-T94A, and RpoD-T208A mutant proteins in which Thr and Ser were replaced with Ala to block its phosphorylation status. Co-incubation of these RpoD mutants with STK exhibited a similar migratory band on the Phos-tag gel, but no such migratory band was noted in the absence of STK (Figure 1D). However, western blot showed that the phosphorylation level of RpoD in RpoD-T208A was completely abolished in the presence of STK compared with RpoD-S93A and RpoD-T94A mutants (Figure 1E), indicating that RpoD is a substrate of STK and Thr-208 is a target site for STK phosphorylation. To assess the conservation of RpoD, we performed multiple sequence alignments of RpoD homologs from different pathogens, the T208 site was conserved across all the examined bacterial species (Figure 1F), indicating its significant evolutionary conservation.

### Phosphorylation of RpoD enhances *S. suis* hemolytic activity

**Figure 2:**
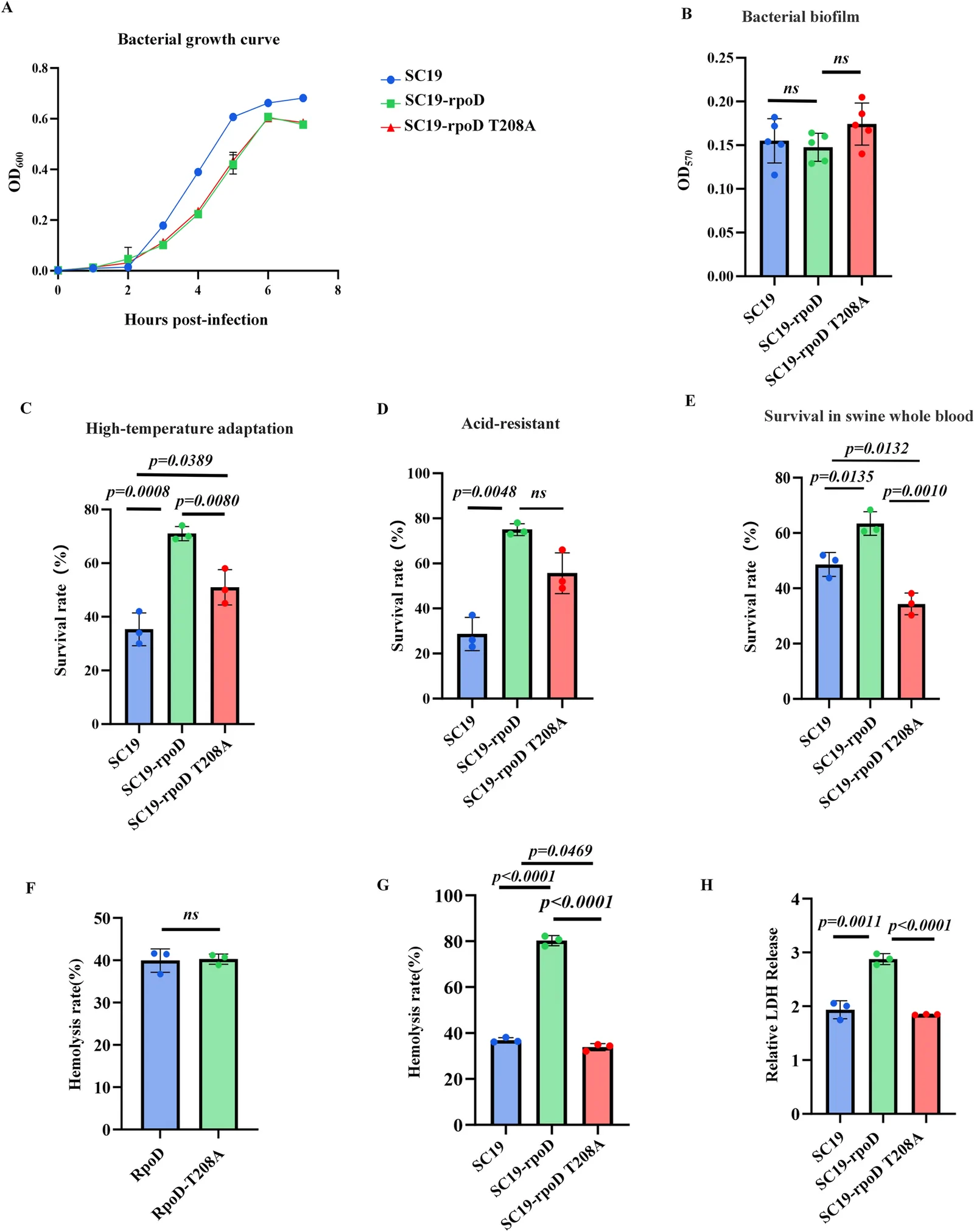
**Phosphorylation of rpoD enhances** ***S. suis*** **hemolytic activity.** **A** Bacterial growth curves of SC19, SC19‑rpoD (overexpressing rpoD), and SC19‑rpoD T208A (phosphorylation‑deficient mutant) in THB plus 10% FBS. **B** Biofilm formation measured by crystal violet staining at OD570. **C** Survival rate after heat stress at 42 °C for 1 h. **D** Survival rate under acidic environment at pH 5.0 for 1 h. **E** Survival in swine whole blood for 3 h. **F**, **G** Hemolytic activity of recombinant proteins and bacterial using swine red blood cells. **H** Relative LDH release in hCMEC/D3 cells infected with SC19, SC19-rpoD, or SC19-rpoD T208A at an MOI of 10 for 4 h. Data are presented as mean ± SD and representative of three independent experiments with triplicate samples per group. The exact *p* values are shown in the figure and a value of *p* < 0.05 was considered statistically significant. ns, not significant.

To elucidate the biological significance of STK-mediated RpoD phosphorylation at Thr-208 in *S. suis*, we characterized the growth profiles, bacterial biofilm, stress tolerance, the survival ability in the blood, and hemolytic activity of SC19-rpoD and SC19-rpoD T208A. The growth of SC19-rpoD and SC19-rpoD T208A appeared slower than that of SC19, but this difference was not statistically significant (*p* > 0.05, Figure 2A). Similarly, the biofilm formation did not exhibit statistically significant differences among SC19, SC19-rpoD, and SC19-rpoD T208A (*p* > 0.05, Figure 2B). Compared with WT SC19, SC19-rpoD exhibited significantly increased survival under high temperature stress (*p* = 0.0008), acidic environment (*p* = 0.0048), and in swine whole blood (*p* = 0.0135) (Figure 2C–E), indicating that RpoD plays a critical role in the stress resistance of *S. suis*. The phosphorylation-blocking mutant SC19-rpoD T208A showed reduced survival under high-temperature stress (*p* = 0.008) and in swine whole blood (*p* = 0.001) relative to SC19-rpoD (Figure 2C and E), while no significant changes were observed in its survival under acidic conditions (Figure 2D), demonstrating that RpoD phosphorylation is required for high temperature adaption and survival in swine blood of *S. suis* but not for its acid resistance. Notably, RpoD T208A mutant protein did not change hemolytic activity (Figure 2F). Compared with WT SC19, SC19-rpoD exhibited significantly enhanced hemolytic activity, while SC19-rpoD T208A exhibited a significantly reduced effect (Figure 2G), indicating RpoD phosphorylation at Thr-208 is essential for regulating the hemolytic activity of *S. suis*.

To further investigate whether RpoD phosphorylation contributes to *S. suis*-induced host cell damage, the relative LDH release was measured in hCMEC/D3 cells infected with SC19, SC19-rpoD, or SC19-rpoD T208A. As shown in Figure 2H, SC19-rpoD induced a significantly higher relative LDH release compared with WT SC19 (*p* = 0.0011), indicating that rpoD overexpression exacerbates bacterial cytotoxicity. In contrast, SC19-rpoD T208A exhibited a significantly reduced LDH release compared with SC19-rpoD (*p* < 0.0001). These results demonstrate that Thr-208 phosphorylation of RpoD is required for *S. suis*-induced LDH release and host cell damage.

### Phosphorylation of RpoD at Thr-208 is required for *sly* transcription

**Figure 3:**
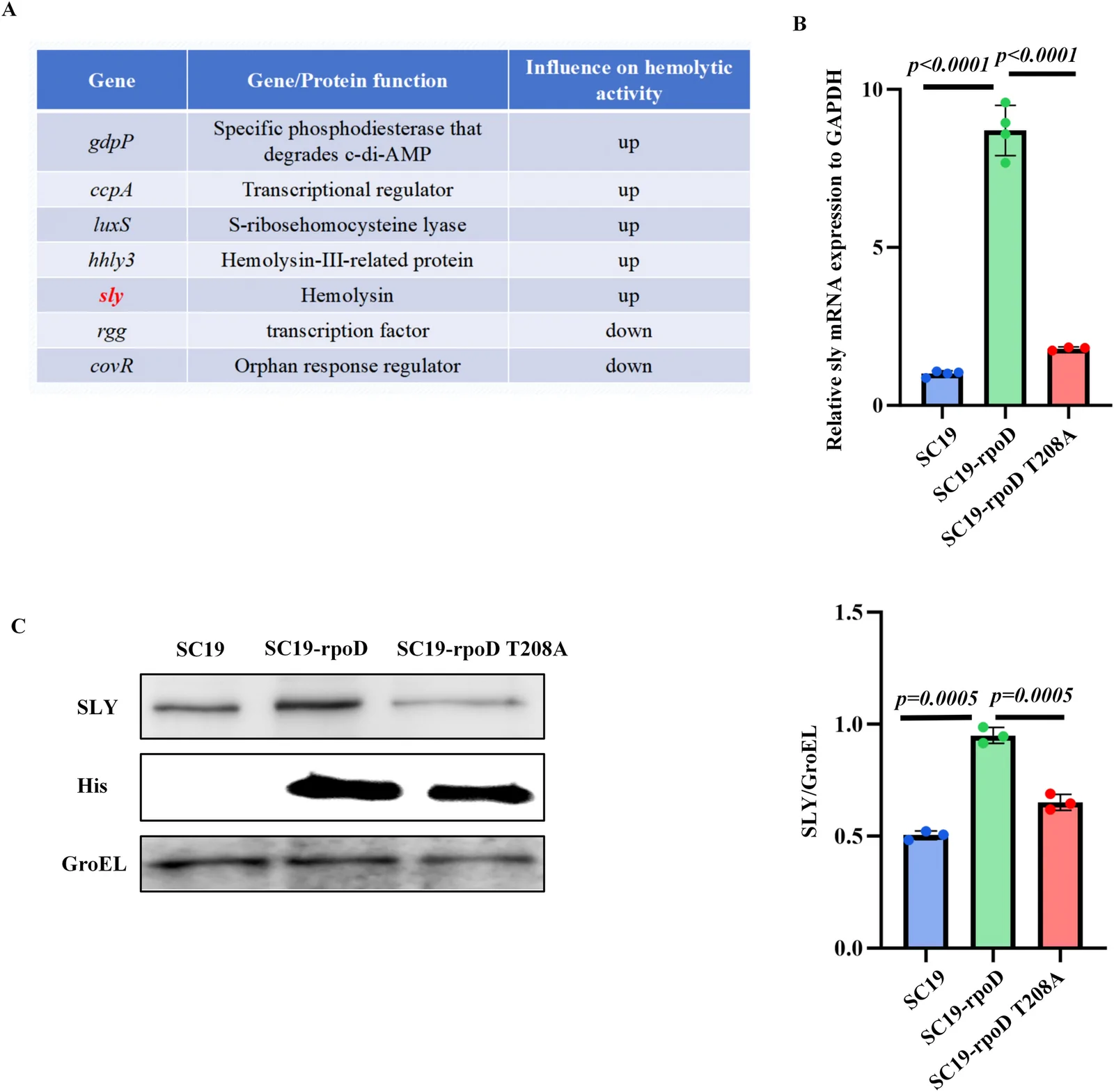
**RpoD phosphorylation upregulates sly transcription and suilysin production.** **A** Hemolysis‑associated genes that have been reported. **B** SLY transcript levels in SC19, SC19‑rpoD, and SC19‑rpoD T208A. **C** Immunoblot analysis of SLY and RpoD in SC19, SC19‑rpoD, and SC19‑rpoD T208A. Data are presented as mean ± SD and representative of three independent experiments with triplicate samples per group. Th exact *p* values are shown in the figure and a value of *p* < 0.05 was considered statistically significant.

The σ^70^ subunit of RNA polymerase has been shown to directly interact with transcriptional activators such as PhoB to promote specific promoter recognition [25]. Since we observed that the phosphorylation of RpoD mediated the hemolytic activity of *S. suis*, we speculated that the phosphorylation of RpoD regulates its hemolytic activity by modulating the transcription of genes associated with hemolytic activity. Hence, we screened genes associated with hemolytic activity including *revSC21* [26], *gdpP* [27]*, ccpA* [28], *luxS* [29], *hhly3* [30], *sly*, *rgg,*, and *covR* (Figure 3A). Among these genes, *sly* is the most closely related to the hemolytic activity of *S. suis* [31]. The results showed that SC19-rpoD significantly upregulated messenger RNA (mRNA) expression (*p* < 0.0001) and protein expression of SLY compared with SC19 (*p* = 0.0008), whereas SC19-rpoD T208A reversed its upregulation (Figure 3B, C), suggesting that phosphorylation of RpoD at Thr-208 is required for *sly* expression.

### Phosphorylation of rpoD enhances *S. suis* pathogenicity

**Figure 4:**
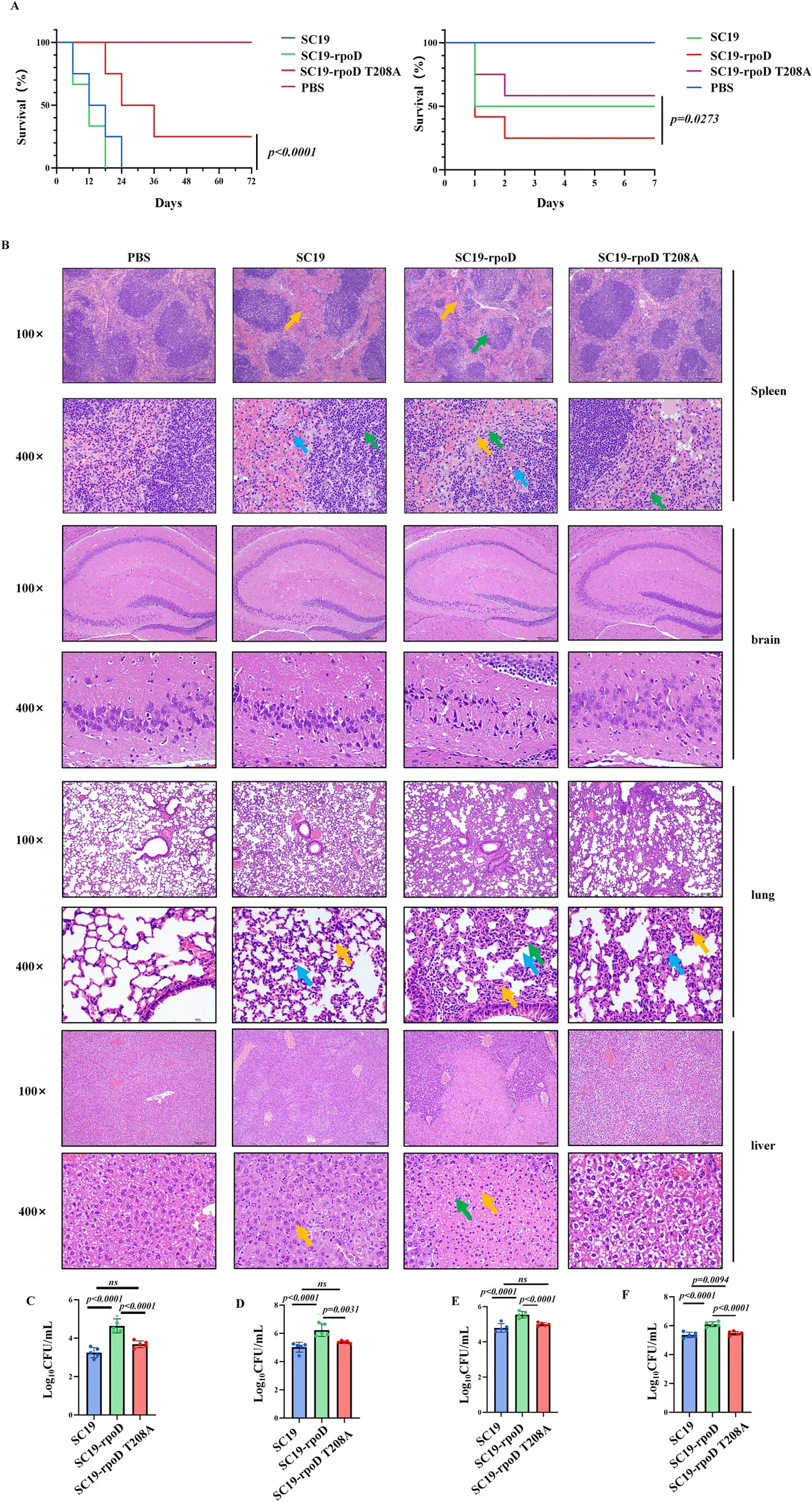
**Phosphorylation of RpoD enhances** ***S. suis*** **pathogenicity.** **A** Survival curves of mice infected intraperitoneally with 2.5 × 10^8^ (left panel) or 5 × 10^7^ (right panel) CFU of SC19, SC19‑rpoD, or SC19‑rpoD T208A (*n* = 10 per group). **B** Representative hematoxylin and eosin (H&E) staining of the spleen, brain, lung, and liver at 24 h postinfection (100× and 400× magnification). Severe pathological lesions were primarily observed in the SC19 and SC19-rpoD groups. The SC19-rpoD T208A and PBS groups showed relatively normal tissue structures with minimal pathological changes. **C**–**F** Bacterial loads in blood (**C**), brain (**D**), spleen (**E**), and lung (**F**) at 24 h postinfection. Data are presented as mean ± SD and representative of three independent experiments with triplicate samples per group. The exact *p* values are shown in the Figure and a value of *P* < 0.05 was considered statistically significant.

To evaluate the effect of STK-mediated phosphorylation of rpoD in bacterial pathogenicity, a mouse infection model was employed. At the high dose (2.5 × 10^8^ CFU) of infection, both WT SC19 and SC19-rpoD caused 100% mortality, while SC19-rpoD T208A infection exhibited significantly increased survival rates (Figure 4A, left panel, *p* < 0.0001). At a lower dose (5 × 10^7^ CFU) of infection, SC19-rpoD still caused higher mortality than SC19, while SC19-rpoD T208A exhibited improved survival (Figure 4A, right panel, *p* = 0.0273). Furthermore, SC19-rpoD induced severe pathological lesions in the spleen, lung, and liver. In the spleen, the lesions were characterized by severe inflammatory cell infiltration (green arrows), splenic sinusoid dilation due to blood stasis (blue arrows), and cellulose-like exudate (yellow arrows). In the lung, the lesions included inflammatory cell infiltration (green arrows), alveolar wall thickening (blue arrows), and inflammatory exudate in the alveolar spaces (yellow arrows). In the liver, the lesions showed inflammatory cell infiltration (green arrows), hepatocellular swelling, and vacuolar degeneration (yellow arrows). The brain exhibited mild edema without obvious inflammatory infiltration (Figure 4B). Consistently, SC19-rpoD led to higher bacterial load in blood, brain, spleen, and lung compared with WT SC19, whereas SC19-rpoD T208A reversed this effect (Figure 4C–F). Taken together, STK-dependent RpoD phosphorylation at Thr-208 potentiates the virulence of *S. suis*.

## Discussion

Sigma factors are common transcription factors in bacteria, which regulate that cell motility, bacterial resistance, and virulence [32]. Here, we identified the housekeeping sigma factor RpoD as a novel substrate of STK in *S. suis* and demonstrated that STK directly phosphorylated RpoD at the Thr-208. This modification is required for *sly* transcription and contributes to bacterial virulence.

STK has been shown to phosphorylate a diverse array of substrates in *S. suis*, thereby regulating bacterial physiological processes, such as cell division, capsular polysaccharide (CPS) synthesis, biofilm formation, and stress resistance [15, 17, 18, 33]. To date, the identified STK substrates comprise CcpS, GntR, FadR, GlmM, EF-P, and SstP1. Phosphorylation of these proteins by STK governs *S. suis* virulence via distinct molecular mechanisms. STK-mediated phosphorylation of CcpS modulates the activity of phosphatase CpsB, which in turn alters the phosphorylation state of CpsD and ultimately regulates CPS synthesis^17^. Phosphorylation of GntR by STK represses transcription of the NADH oxidase gene *nox,* reducing the oxidative stress tolerance and virulence of *S. suis *[34]. STK-dependent phosphorylation of FadR enhances the binding of this protein to the promoter of the arginine deiminase gene (*adi*), upregulating *adi* transcription and consequently enhancing bacterial acid resistance and intracellular survival within macrophages [18]. Our previous work also revealed that STK-catalyzed phosphorylation of EF-P and SstP1 modulates serine protease production and Fe-S cluster biogenesis, respectively, to sustain bacterial survival and virulence under stress conditions [1, 20]. These findings indicate that STK does not regulate bacterial virulence directly through its substrates. Instead, STK-mediated phosphorylation of different substrates acts indirectly by modulating the expression or function of diverse virulence-associated factors and physiological pathways.

Our present study identified that STK phosphorylated RpoD. As the primary housekeeping sigma factor (σ^70^) of RNA polymerase, RpoD is universally conserved across bacterial species and governs the transcription of most constitutive genes for bacterial growth and metabolism [22]. A well-characterized core function of RpoD is the regulation of bacterial heat stress tolerance. It has been reported that RpoD participates in the transcriptional activation of heat shock proteins under high-temperature conditions in *E. coli*, *Vibrio splendidus*, and *Salmonella typhimurium*, enabling bacteria to survive thermal challenges [21, 35, 36]. Similarly, our phenotypic assays also verified that the loss of RpoD phosphorylation markedly impaired the thermotolerance of *S. suis* and survival in the whole blood, further confirming the conserved role of RpoD in bacterial adaptation to environmental stress. However, it has been reported that STK-mediated FadR phosphorylation enhances acid tolerance of *S. suis* by directly binding to the promoter of arginine deiminase (*adi*) and increasing its transcription, which is independent of RpoD phosphorylation, indicating that STK-mediated phosphorylation of different substrates exhibits distinct physiological and virulence‑related phenotypes. Beyond its canonical function in stress resistance, our study demonstrated that STK-mediated RpoD phosphorylation positively upregulated the transcription of *sly* and the production of SLY, a pivotal pore-forming virulence factor of *S. suis*.

SLY is one of the most critical virulence factors of *S. suis *[6]. As a pore-forming toxin, SLY mediates hemolysis, damages host tissue barriers, and triggers excessive inflammatory responses [10]. Despite its critical role in pathogenesis, the regulatory mechanisms governing SLY expression remain unknown. It has been reported that the Ihk/Irr two-component system regulates SLY expression in highly virulent *S. suis* strains, the deletion of the *ihk* and *irr* genes results in a significant reduction in *sly* transcription levels, with reduced bacterial cytotoxicity to a level comparable to that of *sly*-deficient mutant [37]. Deletion of the carbon catabolite control protein A (CcpA), a key regulator of sugar metabolism, also alters the transcriptional level of *sly *[38]. Our study revealed that STK-dependent RpoD phosphorylation enhanced *sly* transcription, suggesting that *sly* transcription is regulated by multiple mechanisms. However, the exact molecular mechanism of the regulation of *sly* transcription remains to be fully defined. Our study provides a novel regulatory mechanism for SLY expression and further enriches the regulatory network of virulence gene expression in *S. suis*.

Although we identified a new substrate of STK and explored how RpoD phosphorylation affected the virulence of *S. suis*, this study still has several limitations. Given that *rpoD* is essential for bacterial growth, a complete *rpoD* knockout strain could not be successfully constructed in the SC19. For this reason, we generated SC19 mutant strains carrying chromosomally integrated pSET2-rpoD-T208A to mimic the phosphorylated and nonphosphorylated status of RpoD, and further examined the effect of RpoD phosphorylation on *sly* transcription and bacterial virulence. We attempted an electrophoretic mobility shift assay (EMSA) to test direct binding of RpoD to the *sly* promoter, but reproducible specific shifts were not obtained; therefore, we do not claim direct promoter binding. RpoD phosphorylation-mediated *sly* transcription may involve other transcriptional regulators, and this hypothesis requires further experimental verification. Notably, whether the specific Thr-208 is phosphorylated in other species remains to be verified. The investigation of RpoD phosphorylation in other pathogenic bacteria could reveal whether this regulatory mechanism is broadly conserved or species-specific. In addition, since RpoD is a global sigma factor, overexpression of *rpoD* or its mutant could have pleiotropic effects, future RNA-sequencing (RNA-seq) will help define the specificity of its downstream pathway.

Collectively, this study identified RpoD as a novel STK substrate that plays a pivotal role in *S. suis* virulence. STK-mediated phosphorylation of RpoD at Thr-208 is required for the upregulation of *sly* transcription and elevated hemolysin production. Loss of this modification significantly attenuated bacterial virulence. Our findings establish a functional link between STK signaling and the SLY pathway. This STK-dependent regulatory pathway may serve as a potential target for the development of anti-*S. suis* strategies.

## Supplementary Information

**Additional file 1. Primers used in paper.**

## Data Availability

No datasets were generated or analyzed during the current study.
