# Supplementary material for: Phosphorylation of RpoD upregulates suilysin and enhances Streptococcus suis virulence
Source: Vet Res. 2026 Oct 30;57:192. doi: 10.1186/s13567-026-01864-1 (PMC13723133; doi:10.1186/s13567-026-01864-1)
Supplement: Supplementary file 1 [file 13567_2026_1864_MOESM1_ESM.doc]

Primers used in paper

| Primers | Sequence (5’-3’) |
| --- | --- |
| pSET2-*rpoD*-F | aggtcgactctagaggatccagaattgcaagctatttatg |
| pSET2-*rpoD*-R | aaacgacggccagtgAATTCtcaatcctcaataaaatctc |
| His-*rpoD*-F | CGAGTGCGGCCGCAAGCTTtcaatcctcaataaaatct |
| His-*rpoD*-R | GGAATTCCATGCACCACCACCACCACCACatgacaaataaaaaa |
| nSTK-F | CGCGGATCCATGATTCAAATCGGTAAGATC |
| nSTK-R | CCGCTCGAGTGTATCAACCTTGTTCCC |
